# A Case Report and a Literature Review of Traumatic Abdominal Aortic Dissection: An Uncommon Complication Following Vertebral Fractures Due to Blunt Trauma

**DOI:** 10.7759/cureus.62899

**Published:** 2024-06-22

**Authors:** Luke Peacock, Syed Farooq Hyder, Hina Pervaiz, Sandeep S Bahia, Salman Naeem

**Affiliations:** 1 Emergency Department, East Kent Hospitals University NHS Foundation Trust, Canterbury, GBR; 2 General Practice Department, Providence Surgery, Bournemouth, GBR; 3 General Practice Department, Brook Lane Surgery, Southampton, GBR; 4 Vascular Surgery Department, East Kent Hospitals University NHS Foundation Trust, Canterbury, GBR

**Keywords:** acute lumbar vertebral fractures, fall, intimal tear, acute traumatic aortic dissection, case report, falling

## Abstract

Aortic dissection is a rare consequence of blunt trauma with potentially fatal consequences requiring prompt identification and management. The most common site for dissection or transection is the thoracic aorta due to anatomical fixation points. Current literature describes four cases of traumatic abdominal aortic dissection with intimal tear associated with vertebral fractures due to falling. We present a 30-year-old gentleman who attended following a fall from a first-floor window. Whole-body computerised tomographic imaging demonstrated superior endplate fractures of L1-L3 vertebral bodies and an acute infra-renal abdominal aortic dissection. He was transferred to the regional tertiary vascular centre and managed conservatively. Clinicians should be conscious of potential aortic dissection in trauma, especially where there is evidence of vertebral fractures. Imaging should be evaluated at the time to specifically exclude such injuries.

## Introduction

Acute aortic syndrome (AAS) describes a syndrome of four acute aortic pathologies: penetrating atherosclerotic aortic ulcer, intramural aortic haematoma, classical aortic dissection and, more recently, incomplete aortic dissection which is characterised by an intimal tear without an intimal flap or haematoma [1]. Classical acute aortic dissection (AAD) is characterised by an intimal flap separating the true and false lumen of the vessel wall. Population studies estimate an annual incidence of AAD of between 2.6 and 7.2 cases per 100,000 people, with a higher incidence in men than in women [2]. Aortic dissection resulting from blunt trauma is a rare occurrence, with 1.79% of patients with blunt trauma experiencing a traumatic aortic dissection (TAD), most commonly in the thoracic aorta [3].

TAD most frequently results from a high energy impact due to rapid deceleration and high energy transfer through tissues including the aorta, such as in high-speed road traffic accidents (RTAs). Up to 80% of patients with TAD die before reaching the hospital and those who reach the hospital alive have a high mortality rate [4]. The major complications of aortic dissection are extension of the tear, thrombosis of the false lumen of the dissection, periaortic haematoma and aortic rupture, which is most often fatal [1]. When TAD does occur, the tear is most likely to occur at the points of greatest hydraulic stress in the right lateral wall of the ascending aorta or the proximal segment of the descending thoracic aorta; rarely does TAD occur in the distal descending aorta [2,5]. The proposed mechanism for the formation of an intimal tear at the aortic isthmus is due to the stretching of the isthmus between the moveable ascending aorta and the fixed descending aorta, attached to the posterior chest wall by the ligamentum arteriosum [5].

TAD due to fall from height is rare as compared to RTAs, with few case reports publishing this potentially life-threatening pathology [5-8]. Consequently, this is an important pathology for clinicians to be aware of to initiate prompt investigation and management to avoid the sequelae that can be rapidly fatal. This case report describes TAD due to vertebral fractures following a fall from height.

## Case presentation

We present a 30-year-old man brought in by ambulance to our emergency department (ED) following a fall from a first-floor window of a building (three metres high). He landed on his feet and subsequently hit his head and chest on the ground. He complained of facial and chest injuries and was found at the scene by an ambulance crew walking unaided.

This gentleman was previously fit and well, with no past medical history of note. On examination, his airway was patent, and he was talking in full sentences. There was equal bilateral chest rise and air entry. Mild tenderness was noted on the right side of the chest wall. Saturations were 98% on room air. He was well perfused peripherally, with a blood pressure of 123/77 and a pulse of 88 beats per minute. Glasgow Coma Scale (GCS) was 15/15, although he was restless lying in bed. A three-centimetre laceration was noted above the left eye. He was moving his neck freely without pain and moving all four limbs. No obvious chest bruising was seen. His abdomen was soft and non-tender on palpation. There was no long bone tenderness although he was complaining of pain in both feet.

**Figure 1:**
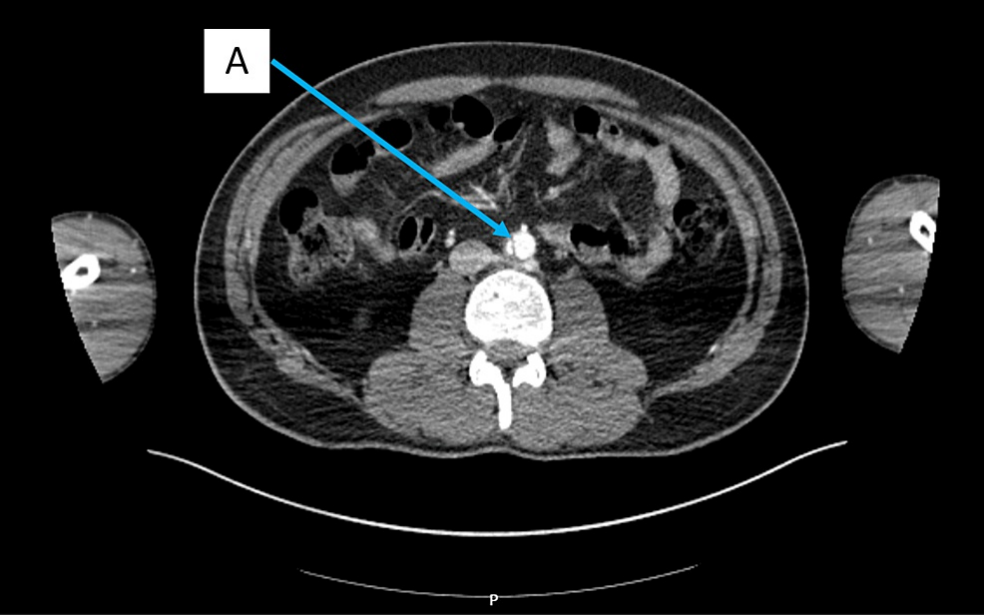
Axial section computed tomography (CT) imaging with contrast showing an intimal tear and aortic dissection (A) in the infrarenal abdominal aorta.

**Figure 2:**
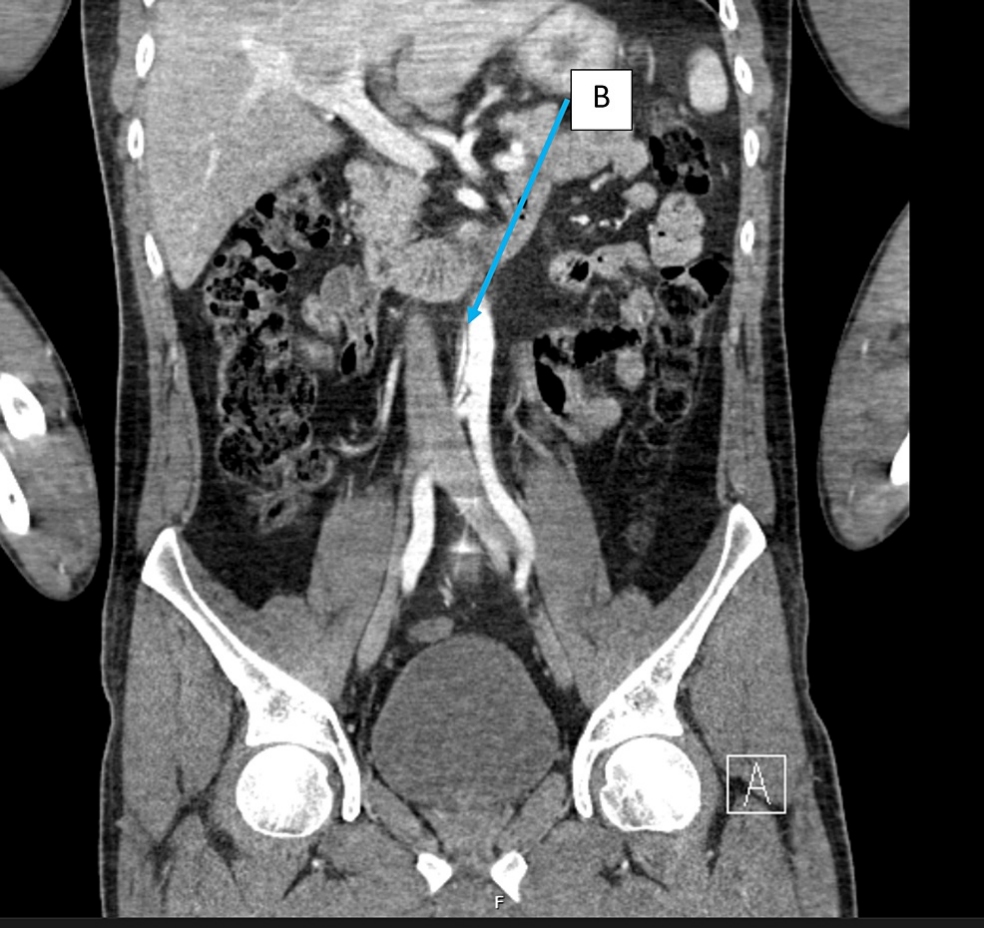
Coronal section computed tomography (CT) imaging with contrast showing an intimal tear and aortic dissection (B) in the infrarenal abdominal aorta.

Full body trauma series computerised tomography (CT) was performed which included a non-contrast CT head, CT cervical, thoracic, lumbar and sacral spine and CT of the chest, abdomen and pelvis with contrast. X-ray imaging was performed on the left knee, both feet and calcanei. CT imaging of the head, chest, abdomen and pelvis showed no acute abnormalities. CT imaging of the spine showed non-displaced fractures of the superior endplates of L1, L2 and L3 vertebral bodies with no retropulsion into the spinal canal. Of note, an intimal flap in the infra-renal abdominal aorta was seen, demonstrating an aortic dissection. This is demonstrated in Figures 1-2. There was no evidence of retroperitoneal haematoma and no active contrast extravasation.

X-ray imaging of the left knee and both feet showed a right medially displaced comminuted extra-articular fracture of the medial process of the right calcaneal tuberosity.

The patient was given analgesia and referred to a tertiary care centre specialising in vascular surgery where he received conservative medical management for the traumatic intimal flap in the infra-renal abdominal aorta. Observations were monitored with a target systolic blood pressure of <110 mmHg to reduce the risk of progression of the dissection [7]. The patient was monitored for signs of mal-perfusion with regular checks of peripheral pulses, abdominal examination and blood tests, particularly for lactate and renal function monitoring. Fluid input and output were closely monitored. Calcaneal tuberosity fractures and L1-L3 superior end-plate fractures were managed conservatively.

He was discharged after seven days of in-patient treatment. He was followed up with a repeat CT angiogram aorta two weeks after discharge which showed a stable unchanged abdominal aortic dissection flap.

## Discussion

Isolated TAD of the abdominal aorta is rare, with dissection most commonly occurring in the ascending aorta and proximal descending thoracic aorta [2,5]. TAD is often a consequence of high-impact and rapid deceleration events, such as RTAs, resulting in high energy transfer through the body [2,5]. Rarely does TAD result due to falling, a mechanism of far lower velocity impact than RTAs [5-8].

Current literature describes a few case reports of patients suffering TAD following a fall with co-existing vertebral fractures. Table 1 shows a summary of case reports of blunt TAD with intimal tears associated with vertebral fractures. Our literature search yielded only four cases of TAD with intimal tear associated with vertebral fractures due to falls, with the lowest height of fall being from five metres. The majority of cases resulted from RTAs.

**Table 1:** Case reports of blunt traumatic aortic dissection with intimal tears associated with vertebral fractures including patient demographics, the type of injury sustained to the aorta, the presence of vertebral fractures and the mechanism of injury.

Citations	Age	Gender (M/F)	Injury to the aorta	Vertebral fracture present	Mechanism of injury
Dorpmans et al. (2023) [7]	66	M	Infrarenal aortic dissection	L1 and T2 fractures	Fall from 7 metres
Yamasaki et al. (2023) [5]	60	M	Dissection at the aortic isthmus	T5-T6 fractures	Fall from 15 metres
Mangold et al. (2022) [9]	4	F	Intimal tear of the infrarenal abdominal aorta	L3-L4 Chance fractures	Road traffic accident (Car)
Chaudhry et al. (2020) [10]	21	M	Thoracic aortic dissection	Thoracic spine fracture (level not specified)	Road traffic accident
Santoro et al. (2018) [11]	37	M	Intimal tear of the abdominal aorta adjacent to the fracture	T12 AO Spine C type fracture	Road traffic accident (Car)
Santoro et al. (2018) [11]	46	M	Intimal tear of thoracic aorta	T11-T12 AO Spine C type fracture	Motorcycle accident with frontal impact
Inaba et al. (2001) [8]	62	F	Intimal flap at the level of L1	L1-L2 shear injury with translation and distraction	Pedestrian hit by a van
Inaba et al. (2001) [8]	52	F	Intimal dissection of the abdominal aorta at level of L1	L1 Chance fracture, L2 burst fracture	Fall from 7 stories
Inaba et al. (2001) [8]	29	M	Intimal tear dissection in the abdominal aorta	L3-L4 Chance fractures	Road traffic accident (Car)
Shweiki et al. (2000) [12]	28	M	Intimal tear 2cm above the bifurcation	L2-L3 fractures	Road traffic accident (Car)
Roth et al. (1997) [13]	21	M	Intimal tear	L2 fracture	Road traffic accident
Poelaert et al. (1995) [6]	32	M	Anterior wall intimal tear at the level of T10	T10 fracture	Fall from 5 metres
Reaney et al. (1995) [14]	15	M	Intimal tears at L3 level	L3 Chance fracture	Road traffic accident
Reaney et al. (1995) [14]	21	F	Intimal tear at L2-3 level	L2 Chance fracture	Road traffic accident
Randhawa and Menzoian (1990) [15]	19	M	Intimal tear of the abdominal aorta	L2-L3 fracture dislocation	Road traffic accident
Stambough et al. (1989) [16]	34	F	Multiple intimal tears from T8 to T11 level	T11-T12 shear fracture	Hit by motorboat whilst swimming
Thal et al. (1971) [17]	38	F	Intimal tear of distal abdominal aorta	L4-L5 fracture dislocation	Road traffic accident
Campbell and Austin (1969) [18]	24	M	Intimal tear of abdominal aorta	L1 wedge fracture	Road traffic accident (Lorry)

The mechanism of aortic injuries due to trauma involving vertebral column fractures is thought to be a combination of high energy transfer through the bodily tissues and stress on the aorta; aortic dissection can occur through shear, rotation, flexion and flexion-distraction movements of the spine due to trauma [14,16]. Studies have demonstrated associations between thoracic spine fractures and aortic rupture as well as between displaced fractures involving the thoraco-lumbar junction (T11-L2) and abdominal aortic injury [11,19,20]. AO Spine classification C-type displaced fractures have been found in more than 70% of cases of TAD with vertebral fractures [11]. Our patient had undisplaced L1-L3 superior endplate fractures. We theorise that in our patient the energy causing vertebral fractures was transferred to the aorta resulting in an intimal tear. This case builds on the evidence of an association between blunt TAD and vertebral fractures, even in low-impact falls. Low-velocity trauma does not exclude TAD. Additionally, this case demonstrates the rare entity of traumatic abdominal, rather than thoracic, aortic dissection occurring without displacement of the vertebral fractures.

## Conclusions

Traumatic abdominal aortic dissection following trauma is a rare entity that clinicians should be aware of especially where there is evidence of vertebral fractures. TAD can be associated with non-displaced vertebral fractures in low-impact mechanisms of injury, such as fall from a low height.
